# The Parameters of Long-Term Humoral Immunity Induced by a Single Injection of the Sputnik Light Vaccine Among Noninfected Volunteers and Those Infected with SARS-CoV-2

**DOI:** 10.32607/actanaturae.27529

**Published:** 2025

**Authors:** F. M. Izhaeva, A. I. Tukhvatulin, A. S. Dzharullaeva, I. V. Dolzhikova, O. V. Zubkova, D. V. Shcheblyakov, N. L. Lubenets, E. A. Smolyarchuk, T. G. Zubkova, A. S. Erokhova, I. M. Cherenkova, K. M. Bukhtin, O. Popova, N. M. Tukhvatulina, B. S. Naroditsky, D. Y. Logunov, A. L. Gintsburg

**Affiliations:** N.F. Gamaleya National Research Center for Epidemiology and Microbiology, Ministry of Health of the Russian Federation, Moscow, 123098 Russian Federation; I.M. Sechenov First Moscow State Medical University, Ministry of Health of the Russian Federation (Sechenov University), Moscow, 119991 Russian Federation; Eco-Safety Medical Center, St. Petersburg, 191119 Russian Federation

**Keywords:** COVID-19, SARS-CoV-2, vaccines, Sputnik Light, vaccine-induced antibody response, serum maturation, hybrid immunity

## Abstract

Although the immunogenicity of clinically approved COVID-19 vaccines remains under intensive investigation, little is still known about the parameters of long-term immune responses. In this paper, we present for the first time the parameters of humoral immunity studied in the phase 1–2 open-label clinical trial of the Sputnik Light vaccine, with a special focus on late follow-up time points (90 and 180 days). For the most accurate assessment of the parameters of humoral post-vaccination immunity (titer and avidity index of antigen-specific antibodies against the RBD domain of SARS-CoV-2), we conducted an additional analysis that allowed us to triage volunteers with immunity formed only in response to vaccination, as well as those with hybrid immunity (infected with SARS-CoV-2 before and after vaccination). The findings indicate that single-shot vaccination with the Sputnik Light vaccine induces a durable (seroconversion 73% on day 180) and mature humoral immunity. Natural immunization as a result of the SARS-CoV-2 infection leads to significant changes in the studied parameters of post-vaccination immunity.

## INTRODUCTION

The outbreak of the COVID-19 pandemic in 2019, caused by the sudden explosive spread of a novel coronavirus known as SARS-CoV-2, significantly affected the health care infrastructures of many countries around the globe [1]. A massive effort consisting of measures for the specific treatment and prevention of COVID-19 was promptly launched. According to the World Health Organization (WHO), over 180 clinical trials were conducted within three years, resulting in the approval of 50 vaccines in different countries [2].

Once the vaccines were cleared for clinical practice, studies aiming to assess their immunogenicity had to be continued. The findings of these studies are needed not only in order to detail the principles that underpin the general functioning of the human immune system, but also to assess and compare the short- and long-term immunogenicity profiles of the current COVID-19 vaccines. It is worth mentioning that conducting, and analyzing the results, of longterm clinical trials is very challenging. An example of such challenges is the inconsistency of the results of long-term clinical trials of the vaccine based on the replication-defective recombinant human adenovirus serotype 26 (Ad26), Ad26.COV2.S (Janssen Vaccines). After comparing the parameters of the humoral immune response between peak values at week 4 and eight months after single-shot administration of the Ad26.COV2.S vaccine, Collier et al. detected an increase in the virus-neutralizing antibody titer (the geometric mean titer (GMT) changed from 1 : 146 to 1 : 629) and a reduction in the titer of IgG antibodies against the receptor-binding domain (RBD) of the SARS-CoV-2 surface glycoprotein (from 1 : 1 361 to 1 : 843) [3]. Yet, another study reported an increase in the titer of anti-RBD IgG antibodies (from 1 : 645 on day 29 to 1 : 1 306 on day 239) and a reduction in the titer of neutralizing antibodies in pseudo-typed virus neutralization assay (from 1 : 272 to 1 : 192) eight months after single-shot immunization with Ad26.COV2.S [4]. Finally, the third long-term clinical trial revealed a decrease in the titer of neutralizing antibodies in pseudo-typed virus neutralization assay (from 1 : 105 to 1 : 41) and a statistically insignificant reduction in the titer of anti-RBD IgG antibodies (GMT, from 1 : 20 447 to 1 : 15 379) during the follow-up period, between 1.5 and 6 months in volunteers subjected to single-shot immunization with Ad26.COV2.S [5].

These inconsistencies in the results could have been caused by the effect of the unregistered COVID-19 infections during the post-vaccination period, which is known to be able to significantly alter the intensity of the immune response [6, 7]. Since all the approved vaccines do not ensure 100% protection against the SARS-CoV-2 infection, clinical trial duration is obviously proportional to the risk of being infected with the coronavirus [8]. In a long-term study, it is impossible to isolate volunteers for the entire follow-up period. Therefore, it is crucial to separate volunteers infected with SARS-CoV-2 during the post-vaccination period from non-infected ones when analyzing the results. Additional challenges may also arise if a small number of volunteers is included in the study. In this situation, the sample size of the group of individuals not infected with SARS-CoV-2 may be insufficient as relates to obtaining statistically significant results.

Previously, we have reported the results of an evaluation of the safety, reactogenicity, and immunogenicity of the Sputnik Light vaccine, which is based on the Ad26 vector carrying the gene encoding the full-length SARS-CoV-2 spike protein, until day 42 of the follow-up period [9]. The objective of this new study was to quantify the changes in the parameters of the post-vaccination humoral immunity in vaccinated volunteers at late follow-up time points (days 90 and 180). For the purpose of obtaining data on auto-immunogenicity of the vaccine, we additionally analyzed serum samples collected from the volunteers (measuring the titers of antibodies specific to the SARS-CoV-2 N protein throughout the study), by selecting a group comprising 59 individuals that had not been infected with the SARS-CoV-2 virus prior to vaccination and remained uninfected throughout the entire period of the clinical trial.

The reported results make it possible to determine the long-term self-immunogenicity of the Sputnik Light vaccine and compare the evolution of humoral post-vaccination immune responses with two groups of volunteers with hybrid immunity: infected with SARS-CoV-2 before (group 2) or after (group 3) vaccination.

## EXPERIMENTAL

**Clinical trial design and procedures**

The phase 1–2 clinical trial designated “An open study on the safety, tolerability, and immunogenicity of the medicinal drug ‘Sputnik Light’ to help prevent the coronavirus infection caused by the SARS-CoV-2 virus” (Protocol No. 06-Sputnik Light-2020) was conducted in 2020 at the medical institution Eco-safety Medical Center (St. Petersburg, Russia). The study was approved by the Local Ethics Committee and authorized by the Ministry of Health of the Russian Federation. The ClinicalTrials.gov identifier is NCT04713488.

The screening procedure was started immediately after informed consent was secured, and it lasted no longer than seven days before study enrollment.

After the screening procedure, six outpatient visits (days 1, 10, 28, 42, 90, and 180 post-vaccination) involving blood collection were arranged. At visit 1 (day 1), the volunteers received a single-dose intramuscular injection of the Sputnik Light vaccine in liquid formulation, developed and manufactured at the N.F. Gamaleya National Center of Epidemiology and Microbiology, Ministry of Health of the Russian Federation, in compliance with Good Manufacturing Practice regulations. The vaccine was based on the recombinant human adenovirus of serotype 26 carrying the gene encoding the full-length SARS-CoV-2 S protein (10¹¹ viral particles per 0.5 mL/dose). A PCR test for SARS-CoV-2 RNA was additionally performed on study days 1, 10, and 28.

**Measuring the titer of IgG total antibodies specific to the RBD of the SARS-CoV-2 S glycoprotein and their subclasses**

Sera were isolated from blood samples by 15-min centrifugation at 4 000 rpm. The sera were subjected to twofold serial dilution, from 1 : 50 to 1 : 102 400. The titer of antigen-specific antibodies was quantified using a kit for enzyme-linked immunosorbent assay of anti-RBD IgG “SARS-COV-2-RBD-IFA-Gamaleya,” manufactured at the N.F. Gamaleya National Center of Epidemiology and Microbiology, Ministry of Health of the Russian Federation (Marketing Authorization No. RZN 2020/10393). The serially diluted serum samples were pipetted onto an antigen-coated plate (100 ng RBD per well) and incubated under stirring (300 rpm, 37°C) for 1 h. After washing with phosphate- buffered saline supplemented with 0.05% Tween-20, HRP-conjugated antibodies specific to human total IgG (NA933-1ML, Cytiva, USA) or IgG1 and IgG4 subclasses (A10648 and A10654, Invitrogen, USA) were added to the plate, and incubation under stirring (300 rpm, 37°C) for 1 h was repeated. After a washing procedure, a tetramethylbenzidine hydrochloride (TMB) solution was added; the plate was incubated in the dark for 15 min, and the reaction was stopped by adding 1 M sulfuric acid. The optical density was measured at 450 nm (OD_450_). The IgG titer was determined as the highest state of dilution of the serum where OD_450_ of the test sample exceeded that of the control serum at the same dilution more than twofold (for each volunteer, his or her own pre-vaccination serum sample was used as their control). If OD_450_ in the serum sample (1 : 50 dilution) was not higher than that in the control serum, the sample was assigned a titer of 1 : 25. All the samples were analyzed in two replicates, and the mean values were determined.

**Determining the avidity index of IgG antibodies specific to the RBD of SARS-CoV-2 S glycoprotein**

Twofold dilutions of sera were pipetted onto plates for the detection of anti-RBD IgG “SARS-COV-2- RBD-IFA-Gamaleya”. One hour later, an equal volume of phosphate-buffered saline or 8 M urea (100 µL) was added to the wells for 10 min. The next procedure was identical to that used when measuring the anti-RBD-IgG titer. The avidity index for each serum was calculated as the ratio between OD_450_ of the well containing the denaturing agent (in the next-to-thelast dilution, being twofold higher than OD_450_ of the control serum in the same dilution) and OD_450_ of the well containing phosphate-buffered saline in the same dilution [10]. All the samples were analyzed in two replicates; the result was determined as the mean value recorded in two replicates.

**Detecting antibodies specific to the SARS-CoV-2 nucleoprotein (N protein)**

Antibodies specific to the SARS-CoV-2 N protein were additionally detected using the *in vitro *ELISA diagnostic kit “K153NG” (XEMA, Russia) according to the manufacturer’s instructions. Sera were diluted 100-fold in a dilution buffer and then added onto a 96-well antigen-coated plate (100 µL per well) in two replicates. The control samples (negative and positive, supplied as part of the kit) were also placed into three additional wells. The plate was incubated at 37°C for 30 min without stirring, rinsed with a wash solution five times, and 100 µL of conjugated secondary antibodies was added into the wells. After incubation and washing according to the procedure described above, 100 μL of the TMB solution was added and the plate was incubated in the dark at room temperature for 25 min. The reaction was then stopped by adding 100 µL of 1 M sulfuric acid per well, and the optical density was immediately measured at 450 nm. For the purpose of interpreting the results, the Cutoff value was found using the following formula: Cut off = X + 0.2, where X is the mean OD_450_ value obtained for Negative Controls 1 and 2. Next, for each sample, the positivity coefficient (PC) was determined using the formula PC = OD_450_ of the sample/cut-off. The result was interpreted as follows: PC > 0.9 is negative; PC > 1.1 is positive. For the samples with 0.9 < PC < 1.1, the assay was repeated using a smaller serum dilution.

**Statistical analysis**

The changes in the parameters within one group over time were compared using the Friedman test with Dunn’s correction. The Kruskal–Wallis test with Dunn’s correction was employed to compare parameters at the same time point between different groups. The correlation was assessed using the Pearson correlation coefficient. The analysis was conducted using the GraphPad 8 and Microsoft Office Excel 2019 software.

## RESULTS

**Fig. 1:**
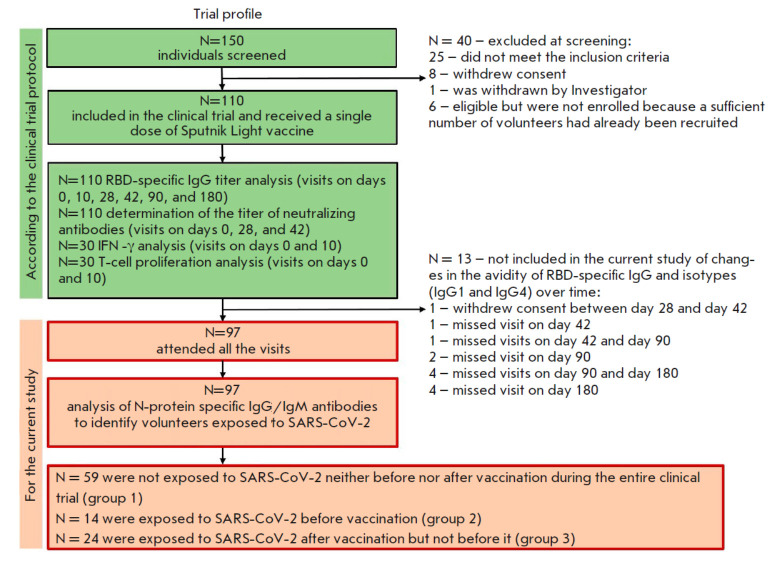
Trial profile and stratification of volunteers into groups

In order to determine the dynamics, including individual ones, of the development of post-vaccination immunity by follow-up day 180, 97 volunteers who had attended all the scheduled blood sampling visits (on days 1, 42, 90, and 180 post-vaccination) were selected (*Fig. 1*).

The serum samples of the volunteers were used to quantify IgG antibodies specific to RBD and the SARS-CoV-2 N protein before and after vaccination. While all the volunteers had negative results in the test for SARS-CoV-2 RNA and ELISA assay for anti- SARS-CoV-2 IgM and IgG at the screening stage, in an earlier publication reporting the results obtained until study day 42, a group consisting of 14 seropositive volunteers with anti-RBD IgG antibodies before vaccination was identified [9]. In this study, antibodies specific to the SARS-CoV-2 N protein were detected in other volunteers using sera collected on days 90 and 180. Hence, to obtain data on the immunogenicity of the Sputnik Light vaccine in the current study, the 97 volunteers were allocated to three groups. The first group comprised volunteers exposed to SARS-CoV-2 neither before nor after vaccination (i.e., those whose humoral immunity parameters depended exclusively on vaccination, n = 59). The immunity of group 2 volunteers was primed with a SARS-CoV-2 infection (n =14) prior to the administration of the Sputnik Light vaccine. Group 3 volunteers (n = 24) had no immunity against SARS-CoV-2 before vaccination but acquired hybrid immunity following a SARS-CoV-2 infection between days 42 and 180 (three participants were exposed to the virus between days 42 and 90; the remaining 21 participants, between days 90 and 180).

**The titer dynamics of RBD-specific IgG antibodies in the serum of non-infected volunteers and those with hybrid immunity**

**Fig. 2:**
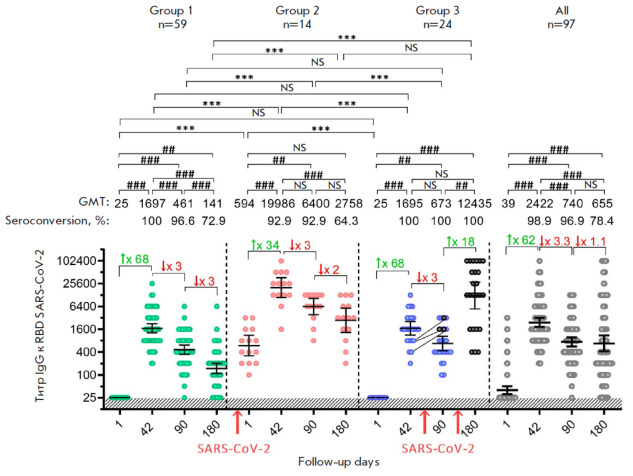
Titer of RBD-specific IgG antibodies in volunteers vaccinated with the Sputnik Light vaccine. The data before (day 1) and on days 42, 90, and 180 post-vaccination for all the analyzed participants (gray dots), as well as those stratified depending on the presence/absence of a SARS-CoV-2 infection, are presented: group 1 – individuals non-infected with SARS-CoV-2 (green dots); group 2 – individuals with SARS-CoV-2 before vaccination (red dots); and group 3 – individuals infected with SARS-CoV-2 post-vaccination (blue dots). Black dots indicate participants infected with SARS-CoV-2 in group 3. The lines between the dots connect the values in the same participant before (day 42) and after infection (day 90). N denotes the number of volunteers in each stratum. Dots show individual data points. Horizontal lines represent geometric mean titers (GMTs); the values are shown above the graph. The percentage of participants (%) who had seroconversion at different time points was defined as a statistically significant, at least fourfold, increase in post-vaccination titer compared to the baseline (day 1). The whiskers represent a 95% confidence interval (CI). The colored numbers with arrows above the square brackets indicate the fold increase or decrease in the GMT compared to the previous time point. The red arrows below the horizontal axis indicate the time of infection with the SARS-CoV-2 virus. Significant differences between different time points within the same group are indicated with hashes: ## *p* < 0.05; ### *p* < 0.005; ### *p* < 0.0001 (calculated using the Friedman test with Dunn’s correction). Statistically significant intergroup differences are indicated with asterisks: * *p* < 0.05; ** *p* < 0.005; or *** *p* < 0.0001 (Kruskal–Wallis test with Dunn’s correction). NS –non-significant difference

An analysis of the titers of RBD-specific IgG antibodies demonstrated that in volunteers with no prior immunity (group 1), vaccination with the Sputnik Light vaccine elicited an abrupt rise in the geometric mean titer (GMT) to 1 : 1 697 on day 42, which then started to prominently decrease, reaching 1 : 461 by day 90 and 1 : 141 by day 180 (*Fig. 2*). In group 2 volunteers with prior immunity against SARS-CoV-2, the GMT at the instant of vaccination was 1 : 594. In this group, immunization triggered the largest increase in the titer of antigen-specific antibodies on day 42 (GMT 1 : 19 986), which then started to decrease at a pace close to that for group 1 (GMT 1 : 6 400 on day 90; GMT 1 : 2 758 on day 180). Group 3 volunteers without prior SARS-CoV-2 immunity (the pre-threshold GMT being 1 : 25 on day 1) exhibited an increase in the humoral immune response on day 42 (GMT 1 : 1 695) similar to that observed in group 1. However, after GMT statistically significantly dropped to 1 : 673 on day 90, it abruptly increased to 1 : 12 435 on day 180. The rise observed on day 180 is attributed to an immunity boost from a prior SARS-CoV-2 infection in all the volunteers in this group (as evidenced by the detection of antibodies specific to the SARS-CoV-2 N protein), whereas on day 90, reduction in the titer of post-vaccination antibodies was not accompanied by significant changes in the GMT of anti-RBD IgG in 3 out of the 24 vaccinated participants (between days 42 and 90). When assessing the results obtained for the entire cohort of 97 volunteers, one can see that the antibody response curve is similar to the response in group 1, where the strongest immunity was observed on day 42 after vaccination, followed by a decline. However, the antibody titers in the overall group were higher than those in group 1 at all the blood collection points, reaching statistically significant differences on day 180 (*p* < 0.002). Furthermore, at later time points, the titer in the overall group decreased more smoothly compared to group 1. In total, the reported results vividly illustrate the effect of the SARS-CoV-2 infection on the intensity of humoral immunity.

**Fig. 3:**
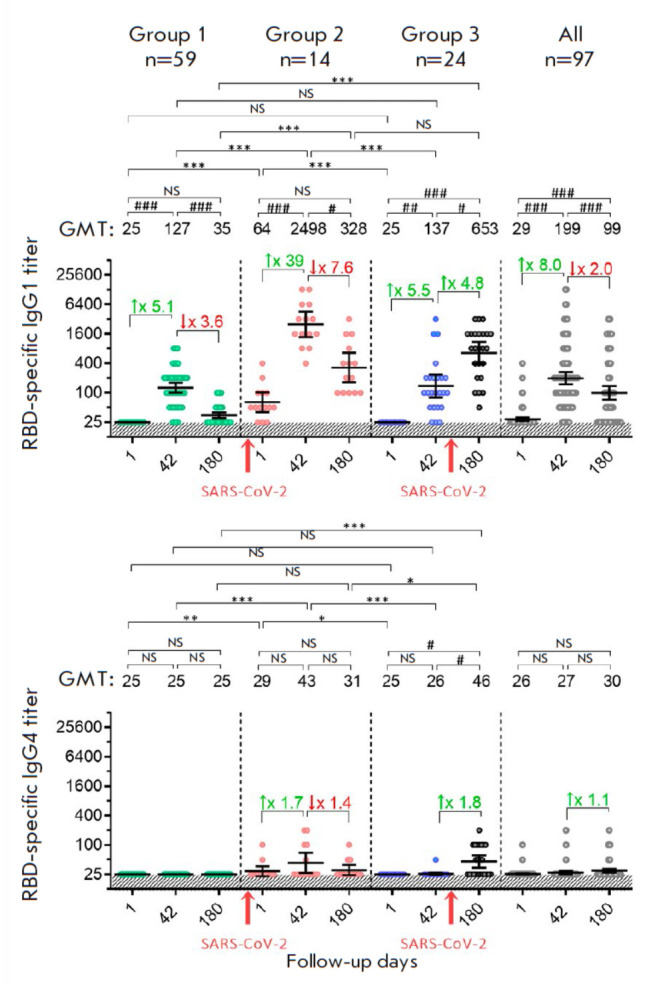
Titer of RBD-specific IgG1 and IgG4 antibodies in volunteers vaccinated with Sputnik Light. The data before (day 1) and on days 42 and 180 after vaccination for all the participants (gray dots), as well as ones stratified depending on the presence/absence SARS-CoV-2 infection: group 1 – individuals not infected with SARS-CoV-2 (green dots); group 2 – individuals infected with SARS-CoV-2 before vaccination (red dots); and group 3 – individuals infected with SARS-CoV-2 after vaccination (blue dots). Black dots indicate participants infected with SARS-CoV-2 in group 3. N denotes the number of volunteers in each stratum. Dots show individual data points. Horizontal lines refer to the geometric mean titers (GMT); whiskers represent a 95% confidence interval (CI). The values are shown above the graph. The colored numbers with arrows above the square brackets indicate the fold increase or decrease in the GMT compared to the previous time point. The red arrows below the horizontal axis indicate the time of infection with the SARS-CoV-2 virus. Significant differences between different time points within the same group are indicated by hashes: # *p* < 0.05; ## *p* < 0.005; ### *p* < 0.0001 (calculated using the Friedman test with Dunn’s correction). Statistically significant intergroup differences are indicated by asterisks: * *p* < 0.05; ** *p* < 0.005 or *** *p* < 0.0001 (the Kruskal– Wallis test with Dunn’s correction). NS indicates non-significant difference

Having detected a prominent rise in total IgG titers after vaccination with the Sputnik Light vaccine, we characterized the changes in the titers of IgG1 and IgG4 antibody subclasses in the analyzed groups. Serum samples were collected at the beginning of the clinical trial (day 1), when the humoral immunity was the strongest in group 1 (day 42), and at the latest follow-up point (day 180) (*Fig. 3*). The IgG1 and IgG4 subclasses were selected because of the differences in their functions and predictive power. IgG1 are the main components in the post-vaccination titer of total IgG antibodies with several defensive functions: antibody-dependent cytotoxicity, phagocytosis, complement activation, and virus neutralization [11, 12]. Meanwhile, the individuals infected with SARS-CoV-2 demonstrated a pronounced rise in the titer of poorly functional IgG4, which allows the virus to evade the defensive responses of adaptive immunity [13]. An analysis of IgG1 antibody titers revealed similar kinetics for total IgG antibodies. Group 1 volunteers with IgG1 titers undetectable on day 1 had a prominent peak (GMT 1 : 127) on day 42 post-vaccination, followed by a reduction on day 180 (GMT 1 : 35). The curve of IgG1 response in group 2 individuals was similar to that of group 1 individuals; the IgG1 titer was maximal on day 42 (GMT 1 : 2 498) and further decreased by day 180 (GMT 1 : 328). In group 3 volunteers, the IgG1 titer was on a gradual increase: it was undetectable on day 1, it increased on day 42 to a level close to that in group 1 (GMT 1 : 137), but it rose to GMT 1 : 653 on day 180, after the hybrid immunity had kicked in. In the overall sample, IgG1 antibody titers on days 42 (GMT 1 : 199) and 180 (GMT 1 : 99) were higher than those in group 1, indicating that inclusion of participants with hybrid immunity can significantly change resulting values. When interpreting the values of IgG4 titers, it is worth noting that this class of antibodies was not detected in group 1. Meanwhile, IgG4 antibodies formed in participants with hybrid immunity (groups 2 and 3) but a statistically significant increase in the IgG4 titer was observed only in group 3 on study day 180. The results support the conclusions that SARS-CoV-2 can trigger the formation of IgG4 antibodies and characterize the inability of the single-shot Sputnik Light vaccine to induce an increase in the IgG4 titer.

**The dynamics of the avidity index of RBDspecific IgG antibodies in the serum of not infected volunteers and those with hybrid immunity**

**Fig. 4:**
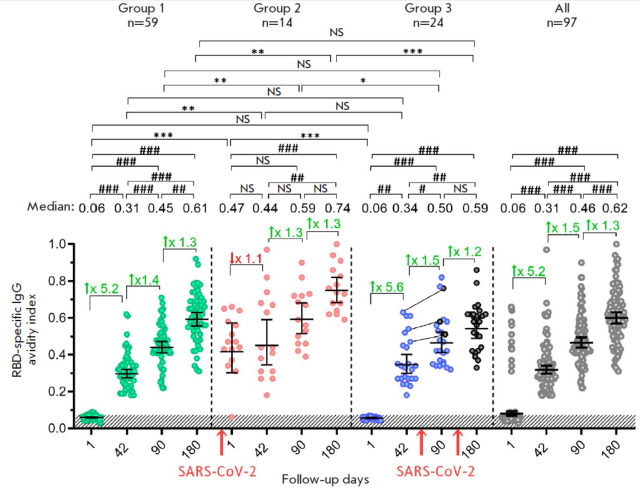
The avidity index of RBD-specific IgG antibodies in volunteers vaccinated with Sputnik Light. Avidity indices are shown before (day 1) and on days 42, 90, and 180 after vaccination for all the participants (gray dots), as well as the ones stratified depending on the presence/absence of the SARS-CoV-2 infection: group 1 – individuals not -infected with SARS-CoV-2 (green dots); group 2 – individuals infected with SARS-CoV-2 before vaccination (red dots); and group 3 – individuals infected with SARS-CoV-2 after vaccination (blue dots). Black dots indicate participants infected with SARS-CoV-2 in group 3. The lines between the dots connect the values of the same participant before (day 42) and after infection (day 90). N denotes the number of participants in each stratum. Dots show individual data points. Horizontal lines represent the geometric mean titers (GMT); the values are shown as black numbers above the graph. Whiskers represent a 95% confidence interval (CI). The colored numbers and arrows above the square brackets indicate the fold increase or decrease in avidity indices compared to the previous time point. The red arrows below the horizontal axis indicate the time of SARS-CoV-2 infection of the volunteers. Significant differences between different time points within the same group are indicated with hashes: # *p* < 0.05; ## *p* < 0.005; ### *p* < 0.0001 (Friedman test with Dunn’s correction). Significant intergroup differences between groups are indicated with asterisks: *p < 0.05; ** *p* < 0.005 or *** *p* < 0.0001 (Kruskal–Wallis test with Dunn’s correction). NS indicates non-significant difference

The measurement of the avidity index of RBDspecific IgG antibodies demonstrated that this parameter continued to gradually increase throughout the entire follow-up period in group 1 volunteers from the minimal values (0.06) on day 1 to 0.61 on day 180 (*Fig. 4*). Group 2 volunteers with prior immunity were characterized by a prominent avidity index of RBD-specific IgG antibodies on day 1 (0.47), which did not show an increase on day 42, but began to noticeably rise starting on day 90 and by day 180 had reached a higher value compared to that for group 1 (0.74). Regardless of exposure to the SARS-CoV-2 infection during the period between day 42 and day 180 of the follow-up period, volunteers in groups 3 and 1 showed similar avidity indices of RBD-specific IgG antibodies. This fact may be indication that the time between the priming immunization and exposure to the boosting coronavirus infection was insufficient [14]. Interestingly, the median avidity index of antibodies for the entire sample did not significantly differ from that in group 1 (unlike for the titers of RBD-specific IgG antibodies) because of the oppositely directed changes in groups 2 and 3. Meanwhile, the overall sample was characterized by significant dispersion of individual data.

**The correlation between the titer and the avidity index of RBD-specific IgG antibodies in the serum of not infected volunteers and those with hybrid immunity**

**Fig. 5:**
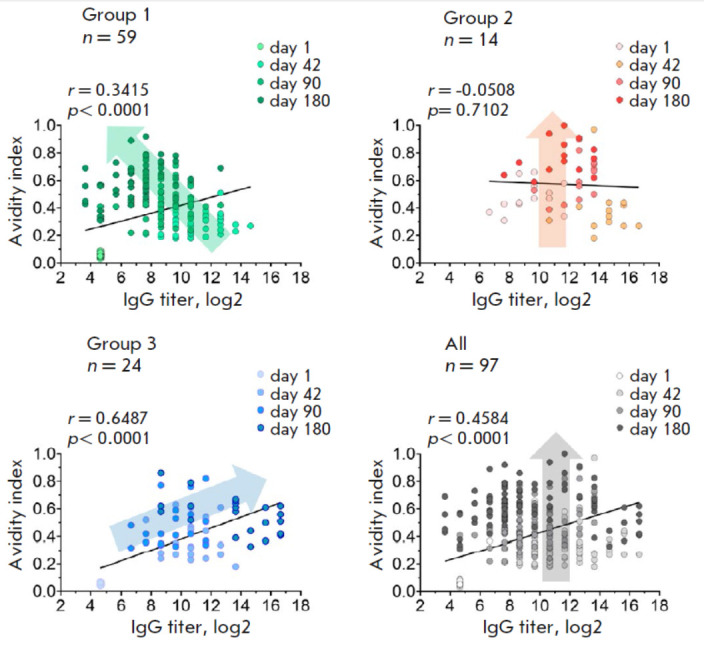
Correlation between the titer and the avidity index of RBD-specific IgG antibodies. Each graph shows the summary data before (day 1) and on days 42, 90, and 180 post-vaccination for all study participants (gray), as well as after stratification depending on the presence/absence of additional immunization due to a SARS-CoV-2 infection: group 1 – individuals not -infected with SARS-CoV-2 (green dots); group 2 – individuals infected with SARS-CoV-2 before vaccination (red dots); group 3 – individuals infected with SARS-CoV-2 after vaccination (blue dots). N is the number of volunteers in each group. Dots represent individual data. Color intensity refers to the study day. Arrows on each graph show the general trend of parameters changing over time. The Pearson correlation coefficient (*r*) and statistical significance (*p*), as well as linear trend line, are shown for each graph

After assessing the changes in the quantitative (the titer) and qualitative (the avidity) parameters of antigen- specific antibodies in volunteers post vaccination with Sputnik Light, we conducted a correlation analysis in groups with indication of the day of blood sample withdrawal (*Fig. 5*). The correlation between the analyzed parameters was found to differ in the course of blood sampling time in all three study groups. Thus, a weak overall correlation between the titer and the avidity index of RBD-specific IgG antibodies (*r *= 0.34) was observed in group 1. It is worth mentioning that the avidity index of serum continued to increase over time, while the titer of antigen-specific antibodies was declining. Interestingly, no correlation between the titer and the avidity index of antibodies (*r *= -0.05, *p *= 0.7102) was observed in volunteers with prior immunity (group 2). The previous COVID-19 infection in group 2 volunteers, which had not increased antibody avidity, apparently, also has a negative impact on serum maturation in the post-vaccination period [15]. The increase in the avidity index by day 180 in group 2 was accompanied by a less prominent decrease in antibody titer compared to group 1. A strong correlation between the titer and the avidity index of RBD-specific IgG antibodies (*r *= 0.65) was revealed in group 3 volunteers infected with SARS-CoV-2 after vaccination, which was reflected in the simultaneous rise in both parameters with time elapsed since vaccination. It is worth emphasizing that the SARS-CoV-2 infection, as well as the time of the event with respect to vaccination, fundamentally alters the evolution of humoral immunity parameters over time. Since the avidity index of total anti-RBD IgG antibodies was increased, all three groups were characterized by different dynamics of anti-RBD IgG titers (shown with arrows in *Fig. 5*). An analysis of the overall sample of volunteers revealed the resulting moderate correlation (*r* = 0.46) between the two parameters, demonstrating that the avidity index of antibodies increased with time, while no noticeable changes in their titer took place.

Hence, our findings demonstrate that natural immunization has a substantial impact on the intensity of the humoral immune response and its maturation with time; thus, it interferes with the self-immunogenicity of the analyzed vaccine product.

## DISCUSSION

The COVID-19 pandemic has set a number of precedents in global science and medicine. A range of vaccines based on different platforms (mRNA, recombinant viral vectors, inactivated or subunit ones, etc.) effectively defending humans against COVID- 19-associated mortality has promptly been developed [16]. Because of the novelty of the pathogen and vaccine products, studies aiming to refine the immune responses that play a crucial role in the development and maintenance of protective immunity in vaccinated individuals need to be continued. In particular, the gained knowledge allows to (1) conduct a comparative analysis of the immunogenicity of vaccines based on different platforms; (2) identify the optimal revaccination time intervals for different population groups, as relates to the new SARS-CoV-2 variants; (3) promptly adapt the antigenic composition of vaccine products in accordance with currently circulating SARS-CoV-2 variants; (4) determine how the developed immune response changes with time after vaccination; and (5) determine the features of the developed immunity in different population groups, etc. The solutions to the aforementioned problems are further complicated by the fact that SARS-CoV-2 remains pervasive in the human population. Undetected exposure to the pathogen can significantly alter the immunogenicity parameters of studied vaccine products [17]. Therefore, particular accuracy is warranted when analyzing the results of clinical trials aiming to assess the immunogenicity of the vaccines, especially those with a long-term follow-up period.

In this paper, we present the results of the phase 1–2 clinical trial to assess the immunogenicity of the Sputnik Light vaccine up to day 180 in the follow-up period. After the clinical trial was completed, we additionally determined whether the participants presented IgG antibodies specific to the SARS-CoV-2 N protein at all time points during the study, by separating the group of volunteers who had been exposed to the SARS-CoV-2 virus neither before vaccination nor throughout the study (group 1), as well as those who had been exposed to the SARS-CoV-2 virus before (group 2) or after vaccination (group 3). Importantly, a small percentage (~ 0.5%) of the Russian population was infected with SARS-CoV-2 at the time the study was initiated (June 17, 2020), which was reflected in our study as a predominance of the percentage of non-infected volunteers (group 1) with respect to the total number of participants (60%) [18]. Therefore, the applied criteria and sample size make it possible to quantify the self-immunogenicity of the Sputnik Light vaccine by a statistical analysis.

The statistically significant drop in the titer of RBD-specific antibodies in group 1 by study day 180 is apparently related to the objective kinetics of the development of the antibody response after a single injection of Ad26-S-based vaccines, which has also been observed by other authors [3, 19]. The titers of antigen-binding antibodies that had been shown in other studies to persist after vaccination with singledose Ad26.COV2.S at late time points may be a result of the influence of some additional stimuli [20, 21]. For example, a dramatic increase in anti-RBD IgG titers, along with a slowly declining titer of total IgG, was clearly demonstrated in volunteers infected with SARS-CoV-2 after vaccination with Ad26.COV2.S [4]. Hence, the lack of a careful selection of volunteers when analyzing the immunogenicity of SARS-CoV-2 vaccines may lead to significant overestimation of the parameters being analyzed.

Like a number of other authors, we have demonstrated that individuals with hybrid immunity (groups 2 and 3) display significantly higher titers of antigenbinding antibodies compared to those in vaccinated volunteers who had not been exposed to SARS-CoV-2 [22]. In the context of persistent population exposure to SARS-CoV-2, the Sputnik Light vaccine appears to be an effective agent for priming and eventually maintaining immunity intensity in individuals that have recovered from COVID-19. Furthermore, the prime-boost revaccination strategy has no advantage in terms of immunogenicity over single-dose administration of the vaccine [23].

The dynamics of antigen-binding antibody titers in volunteers with hybrid immunity indicate that the moment of SARS-CoV-2 infection is important. Thus, in the group of volunteers (group 2) who had been infected before vaccination (according to the titers of antibodies specific to N- and S protein on day 1), the anti-RBD IgG titer increased dramatically, from 594 on day 1 to 19 985 (34-fold) as early as on day 42. Group 3 volunteers infected after vaccination had a less robust rise in anti-RBD IgG titer within the period between days 90 and 180 (18-fold). These results indicate that the interval and/or sequence of vaccination and infection play a pivotal role in the intensity of humoral immunity. Indeed, several studies have confirmed that the efficacy of the boosting stimulus increases with time between immunizations [6, 24]. However, adding a third vaccination within the interval between the first and final vaccination does not significantly increase immunity intensity [25]. The effect of the sequence of exposure to infection and vaccination on immunity intensity has not been identified in detail yet. More convincing data can be provided by clinical studies of the immunogenicity of SARS-CoV-2 vaccines, where the exact time of infection of SARSCoV- 2 volunteers before or after vaccination would be confirmed by laboratory methods (e.g. by PCR tests).

The analysis of IgG antibody subclasses showed that volunteers who had received the single-dose Sputnik Light vaccine lacked IgG4 antibodies until day 180 in the follow-up period, whereas IgG4 antibodies were detected in individuals with hybrid immunity, especially in group 3. This might be caused by differences in immunity against COVID-19 that had developed in response to vaccination and exposure to the SARS-CoV-2 infection. Interestingly, reimmunization with mRNA vaccines (BNT162b2 and mRNA- 1273) can trigger the development of IgG4 antibodies, in contrast to the use of the simian adenovirus-vectored AZD1222 vaccine [26]. Therefore, it seems important to determine whether the detected effect applies to vaccines based on other adenovirus platforms. An analysis of the IgG4 titers in volunteers who had undergone multi-dose vaccination with the Sputnik Light or Sputnik V vaccines would provide an answer to this question.

The avidity of antibodies is an important indicator of the maturation of anti-infection immunity. The avidity of antibodies increases because of the emergence of B-cells with higher affinity antibodies as a result of somatic hypermutation if antigen presentation has sufficient duration and intensity [27].  The SARS-CoV-2 infection is known to slightly increase the avidity of antigen-binding antibodies [28]. Meanwhile, the present study demonstrates that single- shot vaccination with the Sputnik Light vaccine results in a notable rise in the avidity index after immunization.

Along with the increased intensity of humoral postvaccination immunity, exposure to a SARS-CoV-2 infection also significantly affects immune maturation. According to earlier publications, the hybrid immunity in individuals infected with SARS-CoV-2 long before vaccination is characterized by a higher avidity index compared to that in vaccinated volunteers [29]. However, if the SARS-CoV-2 infection occurs shortly after vaccination (group 3), serum maturation is slowed down. This phenomenon can be attributed to the recruitment of new clones of naïve B cells in the proliferative response post-infection (with increasing titers of antigen-specific antibodies), which thus leads to a reduction in the contribution of high-affinity B cells that have already passed the somatic hypermutation stage to the total avidity index (*Fig. 4*). This assumption is reflected in the differing correlations between the titer and the avidity index of RBD-specific IgG antibodies in different groups of volunteers.

## CONCLUSION

This study has for the first time demonstrated the auto- immunogenicity of the Sputnik Light vaccine during a 180-day follow-up period in a clinical trial and assessed the effect of exposure to the SARS-CoV-2 infection before and after vaccination on the parameters of humoral post-vaccination immunity. The findings more accurately characterize the immunogenic properties of the Sputnik Light vaccine, which has been in use in clinical practice since 2020.

## Abbreviations

Ad26: replication-defective recombinant human adenovirus serotype 26
CT: clinical trial
ELISA: enzyme-linked immunosorbent assay
GMT: geometric mean titer
HIV: human immunodeficiency virus
IgG: immunoglobulin G
IgG1: isotype 1 immunoglobulin G
IgG4: isotype 4 immunoglobulin G
IgM: immunoglobulin M
OD: optical density
PC: positivity coefficient
PCR: polymerase chain reaction
RBD: receptor-binding domain
TMB: tetramethylbenzidine
WHO: World Health Organization
